# Prevention of Community-Acquired Pneumonia with Available Pneumococcal Vaccines

**DOI:** 10.3390/ijms18010030

**Published:** 2016-12-25

**Authors:** Nicola Principi, Susanna Esposito

**Affiliations:** Pediatric Highly Intensive Care Unit, Department of Pathophysiology and Transplantation, Università degli Studi di Milano, Fondazione IRCCS Ca’ Granda Ospedale Maggiore Policlinico, 20122 Milan, Italy; nicola.principi@unimi.it

**Keywords:** community-acquired pneumonia, pneumococcal conjugate vaccine, pneumococcal polysaccharide vaccine, pneumococcal prevention

## Abstract

Community-acquired pneumonia (CAP) places a considerable burden on society. A substantial number of pediatric and adult CAP cases are due to *Streptococcus pneumoniae*, but fortunately there are effective vaccines available that have a significant impact on CAP-related medical, social, and economic problems. The main aim of this paper is to evaluate the published evidence concerning the impact of pneumococcal vaccines on the prevention of CAP in children and adults. Available data indicate that pneumococcal conjugate vaccines (PCVs) are effective in children, reducing all-cause CAP cases and bacteremic and nonbacteremic CAP cases. Moreover, at least for PCV7 and PCV13, vaccination of children is effective in reducing the incidence of CAP among adults. Recently use of PCV13 in adults alone or in combination with the pneumococcal polysaccharide vaccine has been suggested and further studies can better define its effectiveness in this group of subjects. The only relevant problem for PCV13 is the risk of a second replacement phenomenon, which might significantly reduce its real efficacy in clinical practice. Protein-based pneumococcal vaccines might be a possible solution to this problem.

## 1. Introduction

Community-acquired pneumonia (CAP) is a common infectious condition in any period of life, with higher impact in resource-limited than industrialized countries [1]. The World Health Organization (WHO) reports >150 million CAP cases each year in children <5 years old, leading to 0.29 episodes per child-year, 20 million hospitalizations, and approximately 2 million child deaths [2]. In industrialized countries, the total number of CAP cases is approximately 4 million, with an incidence of 0.05 episodes per child-year [2,3,4]. Mortality rate is <1 per 1000 per year, with higher values only in subjects with severe chronic underlying diseases [2,3,4]. In adults, it was shown that lower respiratory tract infections (including CAP) are the fourth most common cause of death globally and the second most frequent reason for years of life lost [5]. Within Europe, a recent study has demonstrated an overall annual incidence of 1.07 to 1.2 per 1000 person-years and from 1.54 to 1.7 per 1000 population; incidence further increased with age (14 per 1000 person-years among adults aged ≥65 years) [6].

All the aforementioned reasons explain the burden of CAP on society and the importance of methods able to reduce its incidence and impact [7].

A substantial number of pediatric and adult CAP cases are due to *Streptococcus pneumonia* (*S. pneumoniae*) [8,9], but fortunately there are effective vaccines available that are likely to have a significant impact on CAP-related medical, social, and economic problems [10]. This manuscript aims to study the impact of pneumococcal vaccines on the prevention of CAP in children and adults.

## 2. Pneumococcal Vaccines

Two different pneumococcal vaccines are available in the market: polysaccharide vaccines (PPVs) and conjugate vaccines (PCVs) [11]. In PPV, capsular polysaccharides are purified, but not too much modified, with antigens that remain T-cell independent, whereas the capsular polysaccharides in PCVs are conjugated with a carrier protein and the antigens become T-cell dependent. The majority of the studies comparing the immunogenicity of the two vaccines have shown that PCVs have higher humoral immunogenicity and induce a stronger cell-mediated immunity mainly in younger children who are unable to respond to T-independent antigens [12].

The 23-valent PPV (PPV23) is the only PPV currently marketed. It contains 23 serotypes (i.e., 1, 2, 3, 4, 5, 6B, 7F, 8, 9N, 9V, 10A, 11A, 12F, 14, 15B, 17F, 18C, 19F, 19A, 20, 22F, 23F and 33F) [13].

Different PCVs including various numbers of serotypes (i.e., 7, 9, 10, 11 and 13) and various carrier proteins have been evaluated, but only PCV10 (containing serotypes 1, 4, 5, 6B, 7F, 9V, 14, 18C, 19F and 23F) [14] and PCV13 (containing serotypes 1, 3, 4, 5, 6A, 6B, 7F, 9V, 14, 18C, 19A, 19F, 23F) [15] are currently marketed. These two vaccines have different types of carrier proteins: in PCV10, eight capsular polysaccharides are conjugated to a non-lipidated cell surface lipoprotein (protein D) of non-typeable *Haemophilus influenzae*, and two capsular polysaccharides are conjugated to tetanus or diphtheria toxoid; in PCV13, all serotypes are conjugated to CRM197, a nontoxic mutant of diphtheria toxin. PCV13 replaced PCV7, which included serotypes 4, 6B, 9V, 14, 18C, 19F, and 23F and was used in the first 10 years of this century but was subsequently withdrawn from the market when it became clear that there was a need for vaccines with more pneumococcal serotypes [16], although with a similar immunogenicity [17,18].

Both PCV10 and PCV13 are licensed for use in children from 6 weeks to 5 years of age for prevention of invasive pneumococcal disease (IPD), CAP and otitis media. However, PCV13 has also been licensed for use in older subjects.

## 3. Pneumococcal Vaccine Prevention of CAP in Children

PPV23 is recommended in the USA against infections due to *S. pneumoniae* in children >2 years old with severe chronic underlying diseases associated with an increased risk of complications due to pneumococcal infection [19]. Despite this recommendation, data concerning CAP prevention in pediatric patients have been obtained in children vaccinated with a PCV. Most of the studies in this regard were performed during the period in which PCV7 was used, although recently some epidemiological evaluations carried out after the introduction of PCV10 and PCV13 have reported reliable information on the impact of these new preparations. A systematic comparison of the results of these studies is problematic because of the considerable variations of vaccination schedules, endpoints chosen, population studies, and methodological key points. However, available data indicate that all of the PCVs are significantly effective in reducing the hospitalization rate for all-cause CAP, the incidence of pneumococcal CAP and the risk of death from CAP in vaccinated children, although the impact is different according to the type and number of included serotypes [20,21,22,23,24,25,26,27,28,29,30,31,32,33,34,35,36,37,38,39,40]. Moreover, at least for PCV7, there is clear evidence that the use of the vaccine induces a significant indirect effect, reducing CAP incidence in unvaccinated adults [20,21,22,23,24,25]. Furthermore all suggested schedules of PCV administration yield positive results [41,42,43,44,45], and all PCVs are safe and well tolerated [41,42,43,44,45,46,47,48,49,50,51,52,53,54].

## 4. Heptavalent Pneumococcal Conjugate Vaccine (PCV7)

Several studies, mainly carried out in the USA where PCV7 was implemented in 2000 and the 3 + 1 schedule was used, have evaluated the impact of PCV7 on CAP. Among them, of relevance are the studies carried out by Griffin et al. [20] and Grijalva et al. [21], in which an accurate case definition was used and a long study period was considered. Children <2 years old who were given PCV7 were the subjects who received the greatest benefit from vaccination. However, the benefit tended to decline with increasing age. In particular, Griffin et al. found declines in CAP hospitalization rates of approximately 40% in children <2 years and 12.2% in children aged 2–4 years but not in children aged 5–17 years [20].

Further data showing the positive impact of PCV7 immunization on CAP of both children and adults were reported by Simonsen et al. [22]. Those authors assessed the impact of PCV7 on pneumococcal CAP hospitalization and mortality in all age groups using complete hospitalization data of 10 states in the USA. Compared to a baseline of 1996–1997 through 1998–1999, by the 2005–2006 season, pneumococcal CAP hospitalization and deaths were found to have decreased substantially in all age groups, including a 47% (95% confidence interval (CI) 38–54) reduction in nonbacteremic pneumococcal CAP in infants <2 years old and a 54% reduction (95% CI 53–56) in adults ≥65 years of age. A model developed to calculate the total burden of pneumococcal CAP prevented by infant PCV7 vaccination in the USA from 2000 to 2006 estimated a reduction of 788,838 (95% CI 695,406–875,476) hospitalizations [22]. Ninety percent of the reduction occurred among adults; similar proportions were found in pneumococcal disease mortality prevented by the vaccine. Moreover in the first seasons after PCV introduction, when there were substantial state differences in coverage among <5-year-olds, states with greater coverage had significantly fewer influenza-associated CAP hospitalizations among children, suggesting that PCV7 use could also reduce influenza-attributable CAP hospitalizations. Finally a significant decline in RSV-coded hospitalizations in children aged <1 year was observed following PCV7 introduction (−18.0%, 95% CI: −22.6%, −13.1%, for 2004/2005–2008/2009 versus 1997/1998–1999/2000) [23].

The effectiveness of PCV7 was also evidenced in Europe. In the UK, where PCV7 was added to the national immunization program in September 2006, the incidence of all-cause CAP in 2008–2009 was 11.8/10,000 (95% CI 10.9–12.9), and the hospitalization rate was 9.9/10,000 (95% CI 9.0–10.9) [24]. Compared to 2001, there was a 19% (95% CI 8–29) reduction in the rate of CAP in those aged <5 years, a 33.1% (95% CI 20–45) reduction in the incidence of CAP in those <2 years, and a 38.1% (95% CI 24–50) reduction in hospitalization rates. However for those unvaccinated aged ≥5 years, there was no difference in the incidence of CAP and the hospitalization rate between both surveys.

Despite these and other favorable results, the PCV7 vaccine was abandoned worldwide in 2010 and substituted with new vaccines (PCV10 and PCV13) containing, together with serotypes included in PCV7, some serotypes that, after PCV7 implementation, have emerged as a main cause of pneumococcal disease, slightly but significantly eroding the global impact of the first PCV on IPD [16]. Regarding CAP, it was evidenced that the impact of the replacement phenomenon was limited. Griffin et al. showed that the number of CAP cases in the first 2 years of life and in the age group 2–4 years decreased after the introduction of PCV7, thus limiting concerns that CAP cases caused by serotype replacement would significantly limit the advantage of vaccination [20]. However PCV7 seemed to have a considerable effect on severe CAP because there was an increase in necrotizing CAP and parapneumonic empyema due to serotypes 1, 3 and 19A, which are more likely to cause necrotising CAP [25].

## 5. 10-Valent Pneumococcal Conjugate Vaccine (PCV10) and 13-Valent Pneumococcal Conjugate Vaccine (PCV13)

Epidemiological studies have provided evidence that the new preparations were both effective and safe and could be administered for the prevention of IPD and CAP. Presently it is impossible to state whether there are differences between PCV10 and PCV13 in the prevention of pediatric CAP.

The first results of PCV10 administration were collected in Brazil where the vaccine was introduced in 2010 with a 3 + 1 schedule [26]. Hospitalization rates for CAP among children aged 2–24 months from January 2005 through August 2011 were analyzed. A 23%–29% decline in 3 of 5 cities included in the study one year after the implementation of PCV10 was found. Positive data on CAP incidence following PCV10 implementation were also reported in a study carried out in Chile [27]. This vaccine was introduced in that country in January 2011 with a 3 + 1 schedule without a catch-up vaccination. A population-based nested case-control study using a number of merged nationwide case-based electronic health data registries measured the effectiveness of PCV10 on CAP morbidity and mortality among infants during the first two years after vaccine introduction. A total of 497,996 children were included. The PCV10 effectiveness was 11.2% (95% CI 8.5–13.6) when all CAP hospitalizations and deaths were used to define cases [27]. However the effectiveness increased to 20.7% (95% CI 17.3–23.8) when codes used to identify viral CAP were excluded. Finally effectiveness estimates of pneumonia death and all-cause death were 71.5% (95% CI 9.0–91.6) and 34.8% (95% CI 23.7–44.4), respectively.

A number of studies have analyzed the impact of PCV13 on CAP. In Nicaragua, PCV13 was introduced in December 2010 with a 3 + 0 schedule. Becker-Dreps et al. reported that in that country, the adjusted incidence rate ratio for CAP hospitalization in the vaccine (2011–2012) versus the pre-vaccine (2008–2010) period was 0.67 (95% CI 0.50–0.75) among infants and 0.84 (95% CI 0.74–0.95) among 1-year-old children [28]. Moreover lower rates of health facility visits for CAP among age groups ineligible to receive PCV13 (2 to 4 years old and 5 to 14 years old) were evidenced, providing evidence of herd immunity.

The impact of sequential PCV7 and PCV13 was studied in the USA, where, 2 years after introduction, PCV13 was associated with significant reductions in hospital admissions for all-cause CAP for some children (21%, 95% CI 14–28 in children aged <2 years; 17%, 95% CI 7–27 in those aged 2–4 years) and for empyema (50%, 95% CI 22–68, for children aged <2 years; 46%, 95% CI 21–64, for 2–4 years; 37%, 95% CI 13–54, for 5–17 years) [29]. All-cause pneumonia was significantly reduced in adults aged 18–39 years (12%, 95% CI 6–17) but not for other adult age groups. The vaccine also reduced admissions for invasive pneumococcal CAP and non-invasive pneumococcal or lobar CAP in children and adults, indicating herd protection, although the reduction was significant only in some age groups [29]. Similar positive results were reported by Greenberg et al. in Israel [30]. In that country, PCV7 was introduced in July 2009 and was gradually replaced by PCV13 in November 2010. The PCV impact on the incidence of alveolar CAP, considered a bacterial disease, mainly due to *S. pneumoniae*, was calculated by comparing the incidence of disease in 3 predefined periods: pre-PCV (2002–2008), PCV7 (2010–2011), and PCV13 (2012–2013). The annual incidence of alveolar CAP per 1,000 inhabitants in children <5 years declined from a mean of 13.8 in the pre-PCV period to 11.2 and 7.4 in the PCV7 and PCV13 periods, respectively [30]. The reduction in the alveolar CAP rate in the PCV7 period was mainly observed in outpatients and was more marked in children 12–23 months of age, but in the PCV13 period the rates declined significantly in all age groups among both outpatients and inpatients.

A comparison between the impacts of PCV10 and PCV13 was made by Berglund et al. in Sweden [31]. In that country, PCV7 was implemented in 2009. By 2010, both PCV10 and PCV13 were licensed, and the selection of the vaccine was subject to the County Councils tenders. The introduction of PCV-7 reduced the likelihood of all-cause CAP hospitalizations by 23% among children aged <2 years. In the counties that have substituted PCV7 with PCV13, a trend to a further 18% decrease was observed. On the contrary, where PCV10 was privileged no further decline was reported, suggesting a potential superiority of PCV13 over PCV10 in the prevention of pediatric CAP. Unfortunately the study has some limitations, mainly related to the short time of use of PCVs before evaluation. Herd effects are maximized after only 5–7 years of PCV use and in this study the PCV7 period took only 3 years. Consequently definitive conclusions cannot be drawn. Moreover, lacking data regarding serotypes involved in CAP etiology, it is impossible to state whether this difference must be ascribed to the higher serotype coverage of PCV13 or other undetermined factors.

However, a recent cost-effectiveness analysis of PCVs in preventing CAP in Peruvian children aged <5 years seems to further suggest the superiority of PCV13 [32]. It was shown that PCV10 and PCV13 are more cost-effective than PCV7, but PCV13 prevented more hospitalizations and was more cost-effective than PCV10. Costs per avoided hospitalization were ESD 718 for PCV7, 333 for PCV10, and 162 for PCV13. Finally different serotype composition may play a role in favor of PCV13. This PCV contains serotype 3 and 19A, which are not included in PCV10 and are two of the most common causes of severe CAP and empyema [33]. The importance of serotype 3 is debated. Undoubtedly putative protective levels for this serotype are very high and not achieved in a number of vaccinated subjects [34]. However significant reduction in the risk of IPD due to serotype 3 in children who have received PCV13 has been demonstrated. Considering that protection against CAP probably needs higher antibody concentrations than those needed for IPD prevention, it is likely that a number of cases of this disease associated with serotype 3 infection can occur despite vaccination. It seems to be confirmed by the evidence that in recent years several cases of severe CAP in children vaccinated with PCV13 have been reported [35,36].

Regarding serotype 19A, antibody levels and function after PCV13 have been found significantly higher than those measured after PCV10 [37]. This could lead to greater protection. On the other hand, while introduction of PCV13 has worldwide led to a significant reduction of the 19A pneumococcal infections [38] and carriage [39], this was not always seen after PCV10 use [33]. A clear example of the poor effectiveness of PCV10 against serotype 19A infection is given by the evidence that in New Zealand, where PCV10 was introduced in October 2011, the number of 19A IPD cases reported in the years following PCV10 use was higher than in the years before, leading health authorities of that country to replace PCV10 with PCV13 [40]. However available data and theoretical suggestions must be reevaluated and confirmed, considering also the impact of the replacement phenomenon and vaccine cost, because these factors can be of relevance when making decisions about the choice of the PCV to include in national immunization schedules worldwide.

## 6. Impact of Different Pneumococcal Conjugate Vaccine (PCV) Schedules

All PCVs are usually administered in the first year of life with a primary series of two or three doses given in the first semester of life with a booster dose at approximately one year (i.e., 3 + 1 and 2 + 1 schedules) [41]. A schedule including 3 doses as primary series without a booster dose (3 + 0 schedule) has also been suggested [41]. In children with a severe underlying condition at risk of invasive pneumococcal disease, a booster of PCV13 is recommended between 2 and 6 years of age [41]. Moreover the same vaccine should be administered to at-risk children aged 6–18 years with no history of PCV13 vaccination [41]. As previously mentioned, all suggested immunization schedules are effective in clinical practice. This is well documented by the recent study by Loo et al. [42]. Those authors reviewed studies published from 1994 to 2011 that documented the effects of PCV7 in children of the ages targeted to receive the vaccines. Studying the results of the studies according to the schedule used, they found that the 3 + 1 schedule and the simplified schedules 3 + 0 or 2 + 1 all decrease all-cause CAP [42]. However these data support the WHO recommendation of 3-dose schedules (3 + 0 or 2 + 1) to limit the cost of vaccination and favor compliance in prevention of pneumococcal disease [43]. In resource-limited countries, where the highest incidence of pneumococcal disease and deaths occur before the end of the first year of life, a schedule of early PCV doses (3 + 0 schedule) seems the best solution; in other countries, a schedule with a booster dose may be better against diseases that peak in the second year of life. Whitney et al. [43] showed that the simplified 2 + 1 schedule is the best compromise between effectiveness and cost [44,45].

## 7. Safety and Tolerability of Pneumococcal Conjugate Vaccines (PCVs)

All PCVs are safe and well tolerated [41]. The only problem that emerged during the PCV7 use was the increased risk of severe lung disease, particularly with lung necrosis and parapneumonic empyema [25,44,45,46,47,48,49,50]. Grijalva et al. showed that parapneumonic empyema increased from 3.5 cases per 100,000 children in 1996–1998 to 7.0 cases per 100,000 children in 2005–2007, with the highest increase among children aged 2–4 years [44]. Thomas et al. observed that, although pneumococcal empyema rates remained relatively stable, the roles played by serotypes 1, 3 and 19A significantly increased and they were found in more complicated diseases [45]. Similar results were reported by Byington et al. in the USA [46].

However, although important, emerging pneumococcal serotypes are not the only factors that explain the increase in parapneumonic empyema after the inclusion of PCV7 in the pediatric immunization program. Grijalva et al. observed that a high number of cases in children 2–4 years old were due to streptococci other than *S. pneumoniae* and *Staphylococcus aureus* (*S. aureus*), the rates of which increased by 2.80 and 3.76 times respectively, after the introduction of PCV7 [44]. An increase in *S. aureus* in patients with CAP and empyema has been observed [51]. It has been shown that this may be related to the use of PCV7 because, by inducing a significant reduction in pneumococcal nasopharyngeal colonization for some months, it can favor a temporary increase in *S. aureus* colonization and related diseases [52]. It has been highlighted that the vaccine may disrupt *S. pneumoniae* advantage, which interferes with *S. aureus* colonization by limiting the resources available for its multiplication [53]. However recent epidemiologic evaluations seem to indicate that the risk of empyema development was not increased after introduction of PCV13 [54].

## 8. Vaccine Prevention of Community-Acquired Pneumonia (CAP) in Adults

For more than 2 decades, the prevention of pneumococcal CAP in adults has been based on the administration of PPV23. It was licensed in 1983 to replace an earlier 14-valent formulation without any prelicensure trial evaluating its efficacy against bacteremic pneumonia [13]. Since its introduction on the market in the USA, PPV23 has been recommended for adults ≥65 years. Through 2012, a single dose of PPV23 has been recommended for subjects <65 years old with chronic medical conditions including immunodeficiencies and adults with asthma or who smoke cigarettes [55]. A single re-vaccination with PPV23 five years after the initial dose was recommended before age 65 years for adults with immune compromise or those with functional or anatomic asplenia. In addition, a single dose of PPV23 was recommended for all adults ≥65 years old regardless of previous history of PPV23 [55].

Considering the aforementioned immunological limitations of PPV23, in an effort to protect individuals at risk, immunization with this vaccine should be repeated every 5 years. However the repeated use of PPV23 has been associated with hyporesponsiveness, a phenomenon already described following vaccination with meningococcal polysaccharide vaccines [56,57]. This hyporesponsiveness is characterized by the inability of the re-vaccinated subject to mount an antibody production of at least the same magnitude as the primary response and is ascribed to the immune tolerance induced by the polysaccharide antigens [58,59,60,61]. The magnitudes of hyporesponsiveness seem to differ among pneumococcal serotypes and are related to the number of doses and the intervals between subsequent vaccinations [62]. More than two doses and shortened intervals between vaccinations, particularly in individuals with high levels of circulating antibodies, are associated with a higher risk of reduced antibody production [63]. For these reasons, no more than 2 doses of PPV23, 5 years apart, are recommended.

Despite these immunological problems, PPV23 has been largely used in clinical practice mainly for the prevention of IPD and CAP in adults. Data regarding IPD were satisfactory because they were consistent with significant protection among generally healthy young adults and among the general population of older adults. For example, in a recent meta-analysis of the studies published before August 2015 that independently evaluated the impact of PPV23 in the general population aged 50 years and older, it was shown that the vaccine effectiveness in preventing IPD, including bacteremic CAP, was 50% (95% CI 21–69) for cohort studies and 54% (95% CI 32–69) for case-control studies [64]. However, data regarding efficacy in nonbacteremic CAP, the most common type of pneumococcal CAP, are contradictory. The meta-analysis by Moberley et al. evidenced efficacy against all-cause CAP in low-income (OR 0.54, 95% CI 0.43–0.67) but not high-income countries in either the general population (OR 0.71, 95% CI 0.45–1.12) or in adults with chronic illness (OR 0.93, 95% CI 0.73–1.19) [53]. Conflicting results were also obtained by Huss et al. [52]. When all available studies were considered, those authors found that PPV23 was effective in reducing the risk of presumptive pneumococcal CAP and all-cause CAP (relative risk (RR) 0.64, 95% CI 0.43–0.96, and 0.73, 95% CI 0.56–0.94, respectively). However when only trials of higher methodologic quality were analyzed, no evidence of vaccine protection was demonstrated (RR 1.20, 95% CI 0.75–1.92, for presumptive pneumonia; and 1.19, 95% CI 0.95–1.49, for all–cause pneumonia) [52].

The marginal effect of PPV23 on CAP prevention was described by Kraicer-Melamed et al., who reported for a global population a vaccine effectiveness for CAP of 4% (95% CI −26 to 26) for trials, 17% (95% CI −26 to 45) for cohort studies, and 7% (95% CI −10 to 21) for case-control studies [64]. Conversely a positive effect of PPV23 was reported by other meta-analyses. Among them, one of the most recent was carried out by Diao et al. [65], who reported a weak association between PPV23 administration and prevention of all-cause CAP (RR 0.87, 95% CI 0.76–0.98), especially in individuals aged >65 years and high-risk subjects aged 19–64 years (RR 0.72, 95% CI 0.69–0.94) [65]. Moreover, protective trends of PPV23 in the outcomes of pneumococcal CAP (RR 0.54, 95% CI 0.18–1.65) and mortality due to CAP (RR 0.67, 95% CI 0.43–1.04) were observed.

Practically, convincing evidence that PPV23 can protect adults from CAP is lacking. To provide definitive protection of adults against CAP, the combined use of PPV23 and PCV13 was suggested. Updated guidelines prepared by the Advisory Committee on Immunization Practices (ACIP) for pneumococcal vaccination in adults recommend that pneumococcal vaccine naïve subjects receive PCV13 in series with PPV23 with a time interval of at least one year between doses [5]. The rationale for the association and the sequence was based on immunogenicity studies because no clinical studies evaluating the efficacy of the two vaccines given in series are available. With few exceptions, a single dose of PCV7, the first conjugate pneumococcal vaccine, was generally found able to evoke a higher immune response than a single dose of PPV23, in both immunocompetent and immunocompromised adults [61,66,67,68,69]. Moreover it was evidenced that initial vaccination with PCV13 established an immune state that results in recall anti-pneumococcal responses upon subsequent vaccination with either PCVs or PPV23. In contrast, initial vaccination with PPV23 results in an immune state in which subsequent PPV23 administration yields generally lower responses compared with the initial responses [70,71]. The length of the interval between PCV13 and PPV23 is not definitively established, although some data seem to indicate that a long interval may be required to optimize the immune response to the second immunization after an initial dose of PCV. A comparison of antibody responses after a PCV13-PPSV23 sequence to responses following PCV13 or PPSV23 alone, across two studies with intervals of 1 year and 3–4 years between the two vaccines, indicated that the responses to many serotypes are improved with a 3–4-year interval compared with a 1-year interval [70,71]. However, together with immunogenicity, other factors must be considered when a distance between doses must be established [72]. First of all, practical aspects related to health-seeking behavior of adults to minimize missed opportunities for vaccination must be considered. Moreover the different distributions of pneumococcal serotypes that cause disease according to age might be relevant. A non-negligible amount of pneumococcal infections in adults aged ≥65 years is due to serotypes unique to PPV23 that seem to be less common among younger adults [73]. This could lead to a reduced interval in the oldest individuals and the establishment of longer intervals for younger adults. Moreover, safety reasons can lead to maintaining longer intervals. One study that has compared 2- versus 6-month intervals between PCV13 and PPV23 showed an increased risk of reactogenicity in the group of individuals with close vaccinations [74].

However the use of PCV13 as the first measure to prevent CAP in adults is supported by a recently performed study [75]. A randomized, double-blind, placebo-controlled study named CAPITA (Community-Acquired Pneumonia Immunization Trial in Adults) evaluated the efficacy of PCV13 in preventing the first episodes of vaccine-type strains of pneumococcal CAP, nonbacteremic and noninvasive CAP, and IPD. The study included 84,496 pneumococcal vaccine naïve immunocompetent adults ≥5 years, and the enrolled subjects were followed for an average of approximately 4 years. The vaccine efficacy was found to be 46% in the prevention of first episode vaccine-type CAP, 45% in first episode nonbacteremic and noninvasive vaccine-type CAP, and 75% in vaccine-type IPD. PCV13 was also found to be safe in the studied population, although there were more local reactions and systemic events in the PCV13 group compared to the placebo. Additional endpoints observed during the study included the first episode of nonbacteremic and noninvasive pneumococcal CAP including non-vaccine serotypes and first episode of all-cause CAP. PCV13 efficacy was not found to be statistically significant in either of these groups.

A dose of PCV13 is recommended also for adults that were already given a dose of PPV23. Even in this case, the addition of PCV13 assured higher immune system stimulation with final anti-pneumococcal opsonophagocytic activity titers that were significantly higher for 10 of the 12 common serotypes than those evoked by a second PPV23 administration, although they were generally lower than those obtained when the first vaccination is carried out with PPV23 [55].

However, according to several experts, the inclusion of PCV13 in the immunization schedule of adults remains debatable. The CAPITA study was criticized because funding and support were provided by the manufacturer of PCV13, the study was conducted within one country in a population with little variation in race, and the enrolled subjects were pneumococcal vaccine naïve. Moreover it does not clarify whether PCV13 alone can be as effective as the sequential administration of PCV13 and PPV23 or whether the administration of PCV13 after PPV23 really increases the efficacy of the polysaccharide vaccine [76]. Finally the cost-effectiveness of PCV13 for adults was debated. Considering the results of the study, it was calculated that the required number of subjects aged ≥65 years to be treated to prevent one case of CAP over 3.97 years would be 1030, without any mortality benefit and with very high economic costs [77]. On the other hand, it is possible that the number needed to be treated may be smaller (i.e., the number of preventable infections may be higher) considering the limitations of the study and the fact that the trial population was not representative of the population older than 65 years of age, that at the highest risk of pneumococcal CAP. However the CAPITA trial was designed to estimate vaccine efficacy, whereas to estimate the cost-benefit profile of a vaccine it is mandatory to perform cost-effectiveness analyses [78].

The most important observation regarding the impact of PCV13 in adults rises from the demonstrated herd effect of PCV administration. However, with some exceptions, the use of PCV7 in children was followed by a significant reduction of incidence of both bacteremic and nonbacteremic CAP in adults. Similar results were evidenced after PCV13 introduction [79,80]. These data suggest that if the indirect effects of PCV13 administered to children continue to reduce the burden of vaccine-type disease among adults, the benefit of vaccinating adults with PCV13 is likely to be reduced substantially. The widespread use of PCV7 has almost completely eliminated infections due to serotypes included in the vaccine, and it is highly likely that the same reduction may occur after some years of PCV13 use. A significant reduction in IPD caused by most serotypes included in PCV13 across all age groups has been reported after 3 years of extensive PCV13 use among children [81]. If PCV13 serotypes will become no longer important as a cause of both bacteremic and nonbacteremic CAP, PCV13 administration in adults might become practically useless despite relevant social and economic costs. Stoecker et al. reported that for a cohort of 65-year-old in 2013, the cost of adding PCV13 to the schedule was $62,065 per quality-adjusted life year gained, which rose to $272,621 after 6 years of projected herd protection [82]. This explains why ACIP, in the recommendations for the use of both PCV13 and PPV23, highlights that these recommendations are provisional and must be reevaluated, if needed, in 2018 [55]. In the meantime, the impact of PCV13 and PPV23 on CAP incidence in adult populations should be accurately monitored, and adequately planned studies should be carried out to measure the real importance of PCV13 introduction.

## 9. Conclusions

Available data indicate that PCVs are effective in children, reducing all-cause CAP cases and bacteremic and nonbacteremic CAP cases. Moreover, at least for PCV7 and PCV13, an impact on CAPs among adult people is demonstrated. The only relevant problem for PCV13 is the risk of a second replacement phenomenon, similar to that already reported for PCV7, that might significantly reduce its real efficacy in clinical practice. Protein-based pneumococcal vaccines might be a possible solution for this problem [83]. Unfortunately they will only become available for several years. In the meantime, PCVs must be maintained in the immunization schedule of infants and children and also used to assure protection among older children with risk factors.

Significantly less optimistic is the protection of adult people, particularly those aged ≥65 years with PPV23 alone or in association with PCV13. Real effectiveness of PCV13 and PPV23 in prevention of adult CAP is not precisely defined. It is not clarified whether sequential administration of the two vaccines is significantly more effective than a single administration. Moreover the interval between the two injections is not precisely defined. In addition, the cost-effectiveness of PCV13 use, alone or in combination, must be better established [84].

Further studies in this regard are urgently needed and ACIP’s decision to reevaluate the present recommendations in 2018 in light of new epidemiological evaluations and ad hoc studies seems particularly wise.

## References

[1] Principi N., Esposito S. (2011). Management of severe community-acquired pneumonia of children in developing and developed countries. Thorax.

[2] Rudan I., Boschi-Pinto C., Biloglav Z., Mulholland K., Campbell H. (2008). Epidemiology and etiology of childhood pneumonia. Bull. World Health Organ.

[3] Atkinson M., Yanney M., Stephenson T., Smyth A. (2007). Effective treatment strategies for paediatric community acquired pneumonia. Expert Opin. Pharmacother..

[4] Bryce J., Boschi-Pinto C., Shibuya K., Black R.E. (2005). WHO estimates of the causes of death in children. Lancet.

[5] Lozano R., Naghavi M., Foreman K., Lim S., Shibuya K., Aboyans V., Abraham J., Adair T., Aggarwal R., Ahn S.Y. (2012). Global and regional mortality from 235 causes of death for 20 age groups in 1990 and 2010: A systematic analysis for the Global Burden of Disease Study 2010. Lancet.

[6] Torres A., Peetermans W.E., Viegi G., Blasi F. (2013). Risk factors for community-acquired pneumonia in adults in Europe: A literature review. Thorax.

[7] Raut M., Schein J., Mody S., Grant R., Benson C., Olson W. (2009). Estimating the economic impact of a half-day reduction in length of hospital stay among patients with community-acquired pneumonia in the US. Curr. Med. Res. Opin..

[8] Esposito S., Cohen R., Domingo J.D., Pecurariu O.F., Greenberg D., Heininger U., Knuf M., Lutsar I., Principi N., Rodrigues F. (2012). Antibiotic therapy for pediatric community-acquired pneumonia: Do we know when, what and for how long to treat?. Pediatr. Infect. Dis. J..

[9] Cillóniz C., Ewig S., Polverino E., Marcos M.A., Esquinas C., Gabarrús A., Mensa J., Torres A. (2011). Microbial aetiology of community-acquired pneumonia and its relation to severity. Thorax.

[10] Grabenstein J.D., Klugman K.P. (2012). A century of pneumococcal vaccination research in humans. Clin. Microbiol. Infect..

[11] Dinleyici E.C. (2010). Current status of pneumococcal vaccines: Lessons to be learned and new insights. Expert Rev. Vaccines.

[12] Laferriere C. (2011). The immunogenicity of pneumococcal polysaccharides in infants and children: A meta-regression. Vaccine.

[13] Pneumovax23 Pneumococcal Vaccine Polyvalent. http://www.pneumovax23.com.

[14] European Medicines Agency Synflorix. http://www.ema.europa.eu/ema/index.jsp?curl=pages/medicines/human/medicines/000973/human_med_001071.jsp&mid=WC0b01ac058001d124.

[15] Center for Disease Control and Prevention (2010). Licensure of a 13-valent pneumococcal conjugate vaccine (PCV13) and recommendations for use among children. Advisory Committee on Immunization Practices (ACIP), 2010. MMWR.

[16] Hanage W.P. (2007). Serotype replacement in invasive pneumococcal disease: Where do we go from here?. J. Infect. Dis..

[17] Grant L.R., O’Brien S.E., Burbidge P., Haston M., Zancolli M., Cowell L., Johnson M., Weatherholtz R.C., Reid R., Santosham M. (2013). Comparative immunogenicity of 7 and 13-valent pneumococcal conjugate vaccines and the development of functional antibodies to cross-reactive serotypes. PLoS ONE.

[18] Esposito S., Tansey S., Thompson A., Razmpour A., Liang J., Jones T.R., Ferrera G., Maida A., Bona G., Sabatini C. (2010). Safety and immunogenicity of a 13-valent pneumococcal conjugate vaccine compared to those of a 7-valent pneumococcal conjugate vaccine given as a three-dose series with routine vaccines in healthy infants and toddlers. Clin. Vaccine Immunol..

[19] Nuorti J.P., Whitney C.G., Centers for Disease Control and Prevention (CDC) (2010). Prevention of pneumococcal disease among infants and children—Use of 13-valent pneumococcal conjugate vaccine and 23-valent pneumococcal polysaccharide vaccine—Recommendations of the Advisory Committee on Immunization Practices (ACIP). MMWR Recomm. Rep..

[20] Griffin M.R., Zhu Y., Moore M.R., Whitney C.G., Grijalva C.G. (2013). U.S. Hospitalizations for pneumonia after a decade of pneumococcal vaccination. N. Engl. J. Med..

[21] Grijalva C.G., Nuorti J.P., Arbogast P.G., Martin S.W., Edwards K.M., Griffin M.R. (2007). Decline in pneumonia admissions after routine childhood immunisation with pneumococcal conjugate vaccine in the USA: A time-series analysis. Lancet.

[22] Simonsen L., Taylor R.J., Young-Xu Y., Haber M., May L., Klugman K.P. (2011). Impact of pneumococcal conjugate vaccination of infants on pneumonia and influenza hospitalization and mortality in all age groups in the United States. MBio.

[23] Weinberger D.M., Klugman K.P., Steiner C.A., Simonsen L., Viboud C. (2015). Association between respiratory syncytial virus activity and pneumococcal disease in infants: A time series analysis of US hospitalization data. PLoS Med..

[24] Elemraid M.A., Rushton S.P., Shirley M.D., Thomas M.F., Spencer D.A., Eastham K.M. (2013). Impact of the 7-valent pneumococcal conjugate vaccine on the incidence of childhood pneumonia. Epidemiol. Infect..

[25] Burgos J., Falcó V., Pahissa A. (2013). The increasing incidence of empyema. Curr. Opin. Pulm. Med..

[26] Afonso E.T., Minamisava R., Bierrenbach A.L., Escalante J.J., Alencar A.P., Domingues C.M., Morais-Neto O.L., Toscano C.M., Andrade A.L. (2013). Effect of 10-valent pneumococcal vaccine on pneumonia among children, Brazil. Emerg. Infect. Dis..

[27] Diaz J., Terrazas S., Bierrenbach A.L., Toscano C.M., Alencar G.P., Alvarez A., Valenzuela M.T., Andrus J., del Aguila R., Hormazábal J.C. (2016). Effectiveness of the 10-valent pneumococcal conjugate vaccine (PCV-10) in children in Chile: A nested case-control study using nationwide pneumonia morbidity and mortality surveillance data. PLoS ONE.

[28] Becker-Dreps S., Amaya E., Liu L., Moreno G., Rocha J., Briceño R., Alemán J., Hudgens M.G., Woods C.W., Weber D.J. (2014). Changes in childhood pneumonia and infant mortality rates following introduction of the 13-valent pneumococcal conjugate vaccine in Nicaragua. Pediatr. Infect. Dis. J..

[29] Simonsen L., Taylor R.J., Schuck-Paim C., Lustig R., Haber M., Klugman K.P. (2014). Effect of 13-valent pneumococcal conjugate vaccine on admissions to hospital 2 years after its introduction in the USA: A time series analysis. Lancet Respir. Med..

[30] Greenberg D., Givon-Lavi N., Ben-Shimol S., Ziv J.B., Dagan R. (2015). Impact of PCV7/PCV13 introduction on community-acquired alveolar pneumonia in children <5 years. Vaccine.

[31] Berglund A., Ekelund M., Fletcher M.A., Nyman L. (2014). All-cause pneumonia hospitalizations in children <2 years old in Sweden, 1998 to 2012: Impact of pneumococcal conjugate vaccine introduction. PLoS ONE.

[32] Mezones-Holguín E., Bolaños-Díaz R., Fiestas V., Sanabria C., Gutiérrez-Aguado A., Fiestas F., Suárez V.J., Rodriguez-Morales A.J., Hernández A.V. (2014). Cost-effectiveness analysis of pneumococcal conjugate vaccines in preventing pneumonia in Peruvian children. J. Infect. Dev. Ctries..

[33] Principi N., Esposito S. (2015). The impact of 10-valent and 13-valent pneumococcal conjugate vaccines on serotype 19A invasive pneumococcal disease. Expert Rev. Vaccines.

[34] Andrews N.J., Waight P.A., Burbidge P., Pearce E., Roalfe L., Zancolli M., Slack M., Ladhani S.N., Miller E., Goldblatt D. (2014). Serotype-specific effectiveness and correlates of protection for the 13-valent pneumococcal conjugate vaccine: A postlicensure indirect cohort study. Lancet Infect. Dis..

[35] Syrogiannopoulos G.A., Michoula A.N., Tsimitselis G., Vassiou K., Chryssanthopoulou D.C., Grivea I.N. (2016). Pneumonia with empyema among children in the first five years of high coverage with 13-valent pneumococcal conjugate vaccine. Infect. Dis..

[36] Almeida A.F., Sobrinho-Simões J., Ferraz C., Nunes T., Vaz L. (2016). Pneumococcal pneumonia vaccine breakthroughs and failures after 13-valent pneumococcal conjugated vaccine. Eur. J. Public Health.

[37] Wijmenga-Monsuur A.J., van Westen E., Knol M.J., Jongerius R.M., Zancolli M., Goldblatt D., van Gageldonk P.G., Tcherniaeva I., Berbers G.A., Rots N.Y. (2015). Direct comparison of immunogenicity induced by 10- or 13-valent pneumococcal conjugate vaccine around the 11-month booster in Dutch infants. PLoS ONE.

[38] Waight P.A., Andrews N.J., Ladhani S.N., Sheppard C.L., Slack M.P., Miller E. (2015). Effect of the 13-valent pneumococcal conjugate vaccine on invasive pneumococcal disease in England and Wales 4 years after its introduction: An observational cohort study. Lancet Infect. Dis..

[39] Desai A.P., Sharma D., Crispell E.K., Baughman W., Thomas S., Tunali A., Sherwood L., Zmitrovich A., Jerris R., Satola S.W. (2015). Decline in pneumococcal nasopharyngeal carriage of vaccine serotypes after the introduction of the 13-valent pneumococcal conjugate vaccine in children in Atlanta, Georgia. Pediatr. Infect. Dis. J..

[40] Institute of Environmental Science and Research Ltd (ESR) (2016). Invasive Pneumococcal Disease in New Zealand, 2014. http://www.surv.esr.cri.nz.

[41] Esposito S., Principi N. (2016). Safety and tolerability of pneumococcal vaccines in children. Expert Opin. Drug Saf..

[42] Loo J.D., Conklin L., Deloria Knoll M., Fleming-Dutra K.E., Park D.E., Kirk J., Johnson T.S., Goldblatt D., O′Brien K.L., Whitney C.G. (2014). Methods for a systematic review of pneumococcal conjugate vaccine dosing schedules. Pediatr. Infect. Dis. J..

[43] Whitney C.G., Goldblatt D., O’Brien K.L. (2014). Dosing schedules for pneumococcal conjugate vaccine: Considerations for policy makers. Pediatr. Infect. Dis. J..

[44] Grijalva C.G., Nuorti J.P., Zhu Y., Griffin M.R. (2010). Increasing incidence of empyema complicating childhood community-acquired pneumonia in the United States. Clin. Infect. Dis..

[45] Thomas M.F., Sheppard C.L., Guiver M., Slack M.P., George R.C., Gorton R., Paton J.Y., Simmister C., Cliff D., Elemraid M.A. (2012). Emergence of pneumococcal 19A empyema in UK children. Arch. Dis. Child..

[46] Byington C.L., Korgenski K., Daly J., Ampofo K., Pavia A., Mason E.O. (2006). Impact of the pneumococcal conjugate vaccine on pneumococcal parapneumonic empyema. Pediatr. Infect. Dis. J..

[47] Bender J.M., Ampofo K., Korgenski K., Daly J., Pavia A.T., Mason E.O., Byington C.L. (2008). Pneumococcal necrotizing pneumonia in Utah: Does serotype matter?. Clin. Infect. Dis..

[48] Strachan R.E., Snelling T.L., Jaffé A. (2013). Increased paediatric hospitalizations for empyema in Australia after introduction of the 7-valent pneumococcal conjugate vaccine. Bull. World Health Organ..

[49] Wright N., Hammond P., Morreau P., Hamill J. (2011). Increased incidence of empyema in Polynesian children. N. Z. Med. J..

[50] Singleton R.J., Holman R.C., Wenger J., Christensen K.Y., Bulkow L.R., Zulz T., Steiner C.A., Cheek J.E. (2011). Trends in hospitalization for empyema in Alaska Native children younger than 10 years of age. Pediatr. Infect. Dis. J..

[51] Fedson D.S., Liss C. (2004). Precise answers to the wrong question: Prospective clinical trials and the meta-analyses of pneumococcal vaccine in elderly and high-risk adults. Vaccine.

[52] Huss A., Scott P., Stuck A.E., Trotter C., Egger M. (2009). Efficacy of pneumococcal vaccination in adults: A meta-analysis. CMAJ.

[53] Moberley S., Holden J., Tatham D.P., Andrews R.M. (2013). Vaccines for preventing pneumococcal infection in adults. Cochrane Database Syst. Rev..

[54] Wiese A.D., Griffin M.R., Zhu Y., Mitchel E.F., Grijalva C.G. (2016). Changes in empyema among U.S. children in the pneumococcal conjugate vaccine era. Vaccine.

[55] Centers for Disease Control and Prevention. http://www.cdc.gov/vaccines/hcp/acip-recs/vacc-specific/pneumo.html.

[56] Granoff D.M., Gupta R.K., Belshe R.B., Anderson E.L. (1998). Induction of immunologic refractoriness in adults by meningococcal C polysaccharide vaccination. J. Infect. Dis..

[57] Borrow R., Goldblatt D., Andrews N., Richmond P., Southern J., Miller E. (2001). Influence of prior meningococcal C polysaccharide vaccination on the response and generation of memory after meningococcal C conjugate vaccination in young children. J. Infect. Dis..

[58] Lazarus R., Clutterbuck E., Yu L.M., Bowman J., Bateman E.A., Diggle L., Angus B., Peto T.E., Beverley P.C., Mant D. (2011). A randomized study comparing combined pneumococcal conjugate and polysaccharide vaccination schedules in adults. Clin. Infect. Dis..

[59] Clutterbuck E.A., Lazarus R., Yu L.M., Bowman J., Bateman E.A., Diggle L., Angus B., Peto T.E., Beverley P.C., Mant D. (2012). Pneumococcal conjugate and plain polysaccharide vaccines have divergent effects on antigen-specific B cells. J. Infect. Dis..

[60] Russell F.M., Carapetis J.R., Balloch A., Licciardi P.V., Jenney A.W., Tikoduadua L., Waqatakirewa L., Pryor J., Nelson J., Byrnes G.B. (2010). Hyporesponsiveness to re-challenge dose following pneumococcal polysaccharide vaccine at 12 months of age, a randomized controlled trial. Vaccine.

[61] Dransfield M.T., Nahm M.H., Han M.K., Harnden S., Criner G.J., Martinez F.J., Scanlon P.D., Woodruff P.G., Washko G.R., Connett J.E. (2009). Superior immune response to protein-conjugate versus free pneumococcal polysaccharide vaccine in chronic obstructive pulmonary disease. Am. J. Respir. Crit. Care. Med..

[62] Orthopoulos G.V., Theodoridou M.C., Ladis V.A., Tsousis D.K., Spoulou V.I. (2009). The effect of 23-valent pneumococcal polysaccharide vaccine on immunological priming induced by the 7-valent conjugate vaccine in asplenic subjects with β-thalassemia. Vaccine.

[63] Papadatou I., Piperi C., Alexandraki K., Kattamis A., Theodoridou M., Spoulou V. (2014). Antigen-specific B-cell response to 13-valent pneumococcal conjugate vaccine in asplenic individuals with β-thalassemia previously immunized with 23-valent pneumococcal polysaccharide vaccine. Clin. Infect. Dis..

[64] Kraicer-Melamed H., O’Donnell S., Quach C. (2016). The effectiveness of pneumococcal polysaccharide vaccine 23 (PPV23) in the general population of 50 years of age and older: A systematic review and meta-analysis. Vaccine.

[65] Diao W.Q., Shen N., Yu P.X., Liu B.B., He B. (2016). Efficacy of 23-valent pneumococcal polysaccharide vaccine in preventing community-acquired pneumonia among immunocompetent adults: A systematic review and meta-analysis of randomized trials. Vaccine.

[66] Goldblatt D., Southern J., Andrews N., Ashton L., Burbidge P., Woodgate S., Pebody R., Miller E. (2009). The immunogenicity of 7-valent pneumococcal conjugate vaccine versus 23-valent polysaccharide vaccine in adults aged 50–80 years. Clin. Infect. Dis..

[67] De Roux A., Schmole-Thoma B., Siber G.R., Hackell J.G., Kuhnke A., Ahlers N., Baker S.A., Razmpour A., Emini E.A., Fernsten P.D. (2008). Comparison of pneumococcal conjugate polysaccharide and free polysaccharide vaccines in elderly adults: Conjugate vaccine elicits improved antibacterial immune responses and immunological memory. Clin. Infect. Dis..

[68] Kumar D., Rotstein C., Miyata G., Arlen D., Humar A. (2003). Randomized, double-blind, controlled trial of pneumococcal vaccination in renal transplant recipients. J. Infect. Dis..

[69] Kapetanovic M.C., Roseman C., Jonsson G., Truedsson L. (2011). Heptavalent pneumococcal conjugate vaccine elicits similar antibody response as standard 23-valent polysaccharide vaccine in adult patients with RA treated with immunomodulating drugs. Clin. Rheumatol..

[70] Jackson L.A., Gurtman A., van Cleeff M., Frenck R.W., Treanor J., Jansen K.U. (2013). Influence of initial vaccination with 13-valent pneumococcal conjugate vaccine or 23-valent pneumococcal polysaccharide vaccine on anti-pneumococcal responses following subsequent pneumococcal vaccination in adults 50 years and older. Vaccine.

[71] Greenberg R.N., Gurtman A., Frenck R.W., Strout C., Jansen K.U., Trammel J., Scott D.A., Emini E.A., Gruber W.C., Schmoele-Thoma B. (2014). Sequential administration of 13-valent pneumococcal conjugate vaccine and 23-valent pneumococcal polysaccharide vaccine in pneumococcal vaccine-naïve adults 60–64 years of age. Vaccine.

[72] Pilishvili T., Bennett N.M. (2015). Pneumococcal disease prevention among adults: Strategies for the use of pneumococcal vaccines. Am. J. Prev. Med..

[73] Kobayashi M., Bennett N.M., Gierke R., Almendares O., Moore M.R., Whitney C.G., Pilishvili T. (2015). Intervals between PCV13 and PPSV23 vaccines: Recommendations of the Advisory Committee on Immunization Practices (ACIP). MMWR Morb. Mortal Wkly. Rep..

[74] Miernyk K.M., Butler J.C., Bulkow L.R., Singleton R.J., Hennessy T.W., Dentinger C.M., Peters H.V., Knutsen B., Hickel J., Parkinson A.J. (2009). Immunogenicity and reactogenicity of pneumococcal polysaccharide and conjugate vaccines in Alaska native adults 55–70 years of age. Clin. Infect. Dis..

[75] Bonten M.J., Huijts S.M., Bolkenbaas M., Webber C., Patterson S., Gault S., van Werkhoven C.H., van Deursen A.M., Sanders E.A., Verheij T.J. (2015). Polysaccharide conjugate vaccine against pneumococcal pneumonia in adults. N. Engl. J. Med..

[76] Hayward S., Thompson L.A., McEachern A. (2016). Is 13-valent pneumococcal conjugate vaccine (PCV13) combined with 23-valent pneumococcal polysaccharide vaccine (PPSV23) superior to PPSV23 alone for reducing incidence or severity of pneumonia in older adults? A Clin-IQ. J. Patient Cent. Res. Rev..

[77] Weinberger D.M., Bruhn C.A., Shapiro E.D. (2015). Vaccine against pneumococcal pneumonia in adults. N. Engl. J. Med..

[78] Van Werkhoven C.H., Bonten M.J. (2015). The Community-Acquired Pneumonia immunization Trial in Adults (CAPiTA): What is the future of pneumococcal conjugate vaccination in elderly?. Future Microbiol..

[79] Rodrigo C., Bewick T., Sheppard C., Greenwood S., Mckeever T.M., Trotter C.L., Slack M., George R., Lim W.S. (2015). Impact of infant 13-valent pneumococcal conjugate vaccine on serotypes in adult pneumonia. Eur. Respir. J..

[80] Mendes R.E., Hollingsworth R.C., Costello A., Jones R.N., Isturiz R.E., Hewlett D., Farrell D.J. (2015). Non-invasive *Streptococcus pneumoniae* serotypes recovered from hospitalized adult patients in the United States (2009–2012). Antimicrob. Agents Chemother..

[81] Moore M.R., Link-Gelles R., Schaffner W., Lynfield R., Lexau C., Bennett N.M., Petit S., Zansky S.M., Harrison L.H., Reingold A. (2015). Effect of use of 13-valent pneumococcal conjugate vaccine in children on invasive pneumococcal disease in children and adults in the USA: analysis of multisite, population-based surveillance. Lancet Infect. Dis..

[82] Stoecker C., Kim L., Gierke R., Pilishvili T. (2016). Incremental cost-effectiveness of 13-valent pneumococcal conjugate vaccine for adults age 50 years and older in the United States. J. Gen. Intern. Med..

[83] Esposito S., Principi N. (2016). Strategies to develop vaccines of pediatric interest. Expert Rev. Vaccines.

[84] Liguori G., Parlato A., Zamparelli A.S., Belfiore P., Gallé F., Di Onofrio V., Riganti C., Zamparelli B., Società Italiana di Health Horizon Scanning (SIHHS) (2014). Adult immunization with 13-valent pneumococcal vaccine in Campania region, South Italy: An economic evaluation. Hum. Vaccines Immunother..

